# Realist evaluations in low- and middle-income countries: reflections and recommendations from the experiences of a foreign researcher

**DOI:** 10.1136/bmjgh-2019-001638

**Published:** 2019-10-31

**Authors:** Brynne Gilmore

**Affiliations:** Centre for Global Health, Trinity College Dublin, University of Dublin, Dublin, Ireland

**Keywords:** child health, health systems evaluation, maternal health, other study design

## Abstract

Realist evaluation, a methodology for exploring generative causation within complex health interventions to understand ‘how, why and for whom’ programmes work, is experiencing a surge of interest. Trends indicate that the proliferation in the use of this methodology also applies to research in low- and middle-income countries (LMICs). The value of using realist evaluation for project evaluation is also being noticed by non-governmental organisations (NGOs) and other programme implementers within such contexts. Yet, there is limited exploration of the use of realist evaluations in LMICs, especially their use by foreign researchers. This paper draws on the author’s experience of conducting two realist evaluations across three different sub-Saharan African settings: Mundemu, Tanzania; Kabale, Uganda and Marsabit, Kenya. The realist evaluations were used as an operations research methodology to study two NGO community health programmes. This paper highlights four main challenges experienced by the author throughout the methodological process: (1) power imbalances prevalent during realist interviews, (2) working through translation and what this means for identfying Context-Mechanism-Outcome Configurations, (3) limited contextual familiarity and being an ‘engaged researcher’ and (4) the use or dependence on ‘WEIRD’ theories (i.e. theories based on the study of Western, Educated, Industrialized, Rich, Democratic people) within testing and refinement. Realist evaluation’s enticing and straightforward slogan of finding ‘what works, for whom and why’ is in contrast to the complexity of the methodology used to generate these results (and often to the results themselves). Striking a balance between theory and pragmatism, while adhering to realist ontological underpinnings of generative causation and retroduction, is no easy task. This paper concludes by providing concrete recommendations for those who want to undertake a realist evaluation, with particular attention to cross-cultural settings, in light of the aforementioned challenges. In doing so, it aims to foster improved methodological rigour and help those engaging in this research methodology to work towards more appropriate and contextually relevant findings.

**Summary box**

- Realist evaluations are increasingly being used in low- and middle-income countries and cross-cultural settings to study complex health interventions.
- Methodological challenges of using realist evaluation in such circumstances have yet to be thoroughly explored.
- Issues of power imbalance, using translators, limited contextual familiarity and utilisation of inappropriate theories may be prevalent in such cases.
- Highlighting methodological challenges of using realist evaluation in low- and middle-income countries can ensure methodological rigour.

## Introduction

Realist evaluation, a methodology for understanding complex health interventions, is a form of theory-driven evaluation which acknowledges that interventions and their outcomes are subject to contextual influences. As such, a realist evaluator’s duty is to understand ‘how, why, for whom and under which conditions’ interventions work.^1^ To do this, realist evaluators identify Context-Mechanism-Outcome Configurations (CMOCs).^1^ These configurations describe how specific contextual factors (C) work to trigger particular mechanisms (M), and how this combination generates outcomes (O), thus introducing the concept of generative causality (the idea that mechanisms operate in specific contexts to generate outcomes). By exploring these configurations of change, realist evaluations aim to understand how a programme is expected to work within specific contexts and what conditions may hinder successful outcomes,^1 2^ in order to produce policy relevant findings that can be transferred across settings and contexts.^3 4^

**Figure 1:**
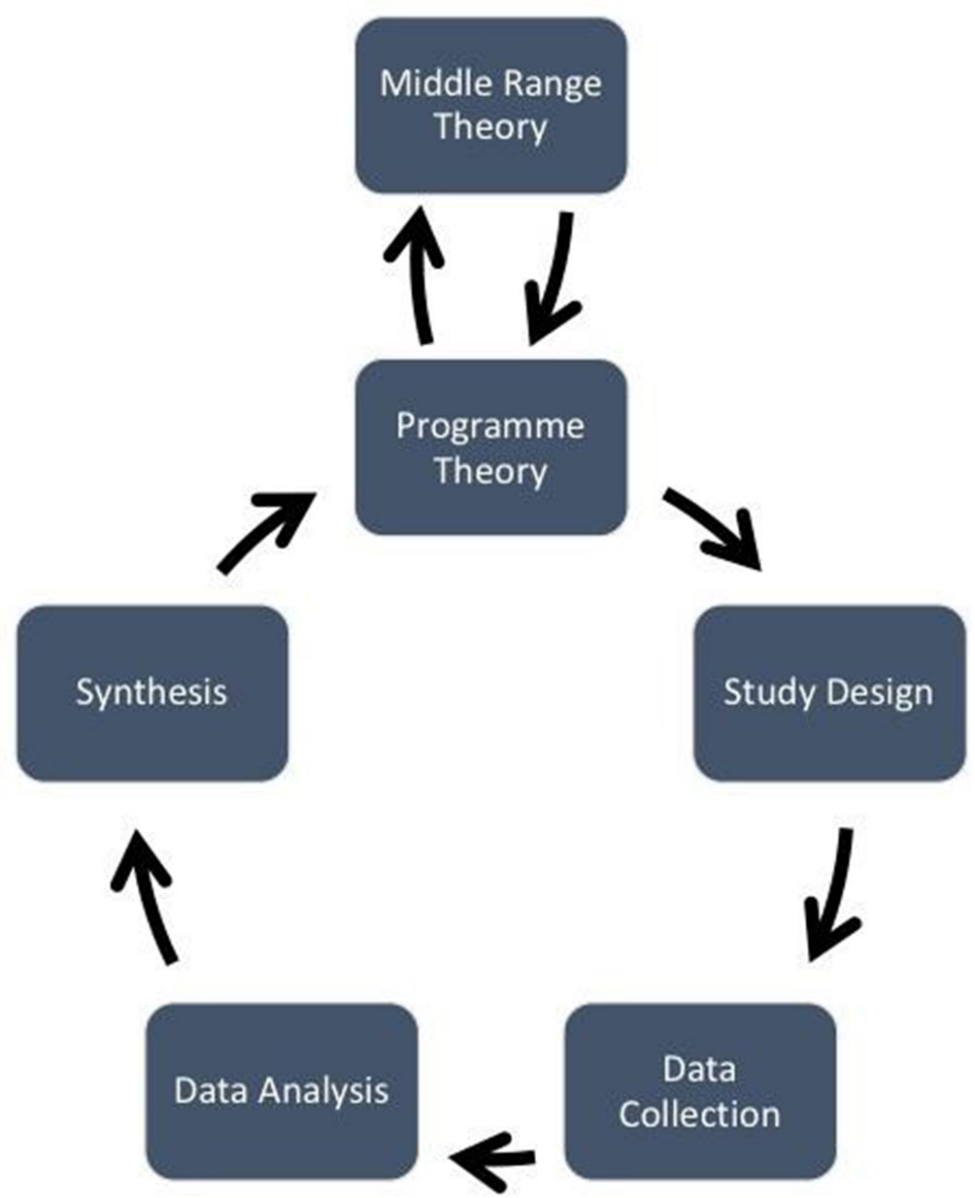
Realist evaluation cycle. Source: Mukumbang *et al* (2016).^48^

The process of conducting a realist evaluation follows a cycle, as shown in figure 1. The evaluation will usually start and end with a theory or hypothesis about how the programme works. The study design, data collection, data analysis and data synthesis all contribute to testing and refining that theory. A multimethod evidence base and inclusion of various stakeholder groups as research participants is usually recommended,^5 6^ though not always necessary. Regardless of the data source, it is important that data collection and analysis work to refine the programme theory or theories by identifying generative causality. Data analysis should be retroductive, which refers to ‘the identification of hidden causal forces that lie behind identified patterns or changes in those patterns’.^7^ Retroduction includes the researcher’s insights and can use both deductive and inductive reasoning to identify generative causation.^8^ By applying principles of generative causation and retroduction, ‘engaged realist’ researchers^6^ can elicit CMOCs and refine contextually relevant programme theories that explain how, why and for whom, interventions work (or do not work).^9^

The Realist And Meta-narrative Evidence Synthesis: Evolving Standards (RAMESES) II Project has produced important guidance to support researchers in conducting and reporting realist evaluations.^10 11^ Their development was informed by published realist evaluation studies, the large majority of which occurred in high-income countries, and a Delphi study with 33 participants, all of whom were affiliated with institutions in high-income countries.^10^ Considerations for the use of this methodology within a wide variety of contexts is an important endeavour moving forward.

While realist evaluation offers an alternative approach to study complex health interventions, they are not without challenges, some of which can be attributed to a lack of procedures and precedent regarding its practice.^12^ A concern most frequently highlighted by realist researchers is the difficulty of defining ‘mechanisms’, and distinguishing them from ‘context’, both of which are tightly intertwined.^3 5 12 13^ It is also possible to find multiple combinations of mechanism and context which can bring about a variety of outcomes.^14^ There is limited guidance on how to code for and identify CMOCs and theories,^8^ which requires a substantial amount of time,^15^ researcher reflection and creativity.^16^ Striking a balance between theory and pragmatism, while adhering to realist ontological underpinnings of generative causation and retroduction, is no easy task and often requires researchers to fall back on their own experience.

## Realist evaluation within low- and middle-income countries by foreign researchers

Realist evaluations are increasingly being employed within low- and middle-income countries by academics, non-governmental organisations (NGOs) and other programme implementers as evidenced by the rapid growth of the number of published realist evaluations from these countries.^17–25^ There remains, however, little precedent for its use within these countries. While the principles of realist evaluation should not change, challenges and implications for the use of this methodology in various settings must be acknowledged and carefully considered. This may be especially pertinent when the researchers are not from the settings where the work is taking place, and questions need to be asked on what influences an unfamiliar context or stakeholder groups has on a realist researcher’s ability to be ‘engaged’.

The main methodological challenges experienced by the author include the influence of power imbalances within data collection, use of translators for realist interviews, limited contextual understanding or engagement and identifying appropriate programme theories reflective of the context. Sharing lessons from practice and ensuring these important methodological discussions occur is an important step to move realist evaluation forward within these contexts. Several authors have shared reflections or recommendations for realist evaluation from projects that were conducted within LMICs;^26 27^ but their focus was not on the cross-cultural challenges. This paper therefore contributes towards the further development of this traction-gaining methodology and aims to inform future work as well as contribute new knowledge on how to conduct realist evaluations within cross-cultural contexts through experiences of a non-local researcher in an LMIC.

Findings and discussions presented in this paper arose from two mixed methods realist evaluations of community health interventions implemented by international NGOs. The first is of a Maternal, Newborn and Child Health (MNCH) intervention within Tanzania and Uganda that consisted of 6 case studies, with a total of 213 participants (116 in Uganda and 97 in Tanzania). This involved doing realist interviews and/or focus group discussions (FGDs) using realist techniques with research participants, majority of whom were rural community members. In total, 18 FGDs were held (three within each case study) and 38 individual interviews, 21 in Uganda and 17 in Tanzania. Interviews were conducted in English, Kiswahili (Tanzania) or Rukigian (Uganda).

The second study, currently ongoing, aims to explore community engagement for health behaviour change. It uses four mixed-method case studies across four different subcounties within Marsabit, Kenya. Data collection using a variety of methods including quantitative surveys, FGDs, semistructured interviews, key informant interviews, document reviews and observations, are being conducted at three different iterative timepoints throughout a 12-month intervention (baseline, midterm and endline). Non-English interviews are conducted in either Borana, Gabra, Rendille or Samburu. To date, 398 surveys, 10 FGDs, 35 interviews and 9 months of programme observations have been completed. The discussions that follow are based on the experience of conducting these two evaluations.

Both studies had coinvestigators from the country in which the research occurs. Coinvestigators specifically supported the design of the study and data collection tools and the analysis. Data collection was performed in each context by myself and a research assistant who provided translation. For the first study (Uganda and Tanzania), research assistants took part in a 3-day training. The training for research assistants for the second study in Kenya took place over 2 days with a 1-day refresher training before each additional round of data collection. Training in both studies included: the intervention of interest, the research study, realist methodology, realist data collection and interviewing and the interview guides. For any interview not conducted in English, the research assistant conducted the interview or FGD. I was present for all qualitative data collection within both studies.

The research studies this paper draws from are not without limitations. Notably, in the first study, there was no theory gleaning phase and initial programme theories (IPTs) were quite specific. Both studies had in-country researchers as coinvestigators who understood the local context; however, none of these were realist researchers. Additionally, all research assistants had experience in qualitative data collection, but none had previous experience in realist evaluation or realist interviews. Furthermore, while I had exposure to realist methodologies prior to the first study, this was my first complete realist evaluation and as such, learnt important lessons along the way—many of which I share below.

## Challenge 1: power imbalances

The topic of power imbalances between researchers and research participants is extensively discussed in qualitative research methodologies.^28 29^ Due to the nature of the researcher-participant relationship, power imbalances may frequently and easily come into play, which can result in controlling or constraining views and respondent bias through enforcing opinions.^29^ Power is noted to be influenced by a number of factors, including educational or professional backgrounds, socioeconomic status, gender and ethnic identities.^30^ This imbalance of power may be exacerbated in less resourced contexts or cross-cultural research situations, is relevant in many global health research projects,^31^ and requires particular attention when working with marginalised populations.^32^ Power discrepancies between researcher and research participant that influence participant responses were also apparent while conducting realist interviews. However, this issue has yet to be thoroughly explored within realist evaluation methodology.

The realist interview is a method for data collection which uses semi-structured, theory-driven interviews to ‘confirm, refute or refine’ theories about how the programme works.^6 9 33^ Using the ‘teacher-learner approach’, the realist interview entails having the interviewer teach the research participant the theory being explored, and after learning the theory, the participant then teaches the researcher their ideas on that theory.^6^ A detailed discussion on the realist interview can be found in Pawson’s work ‘*Theorizing the interview’*.^34^ More recently, Manzano’s^33^ publication reviews interview techniques within realist evaluation noting that ‘surprisingly, qualitative interviewing is treated as unproblematic with little attention given to fieldwork processes or the act of the interview itself’.

The interview process conducted as part of the studies outlined here was found to be problematic for some participant groups (exception being key informants mostly outside of the community setting, like programme managers). Likely exacerbated by the rural, community-based setting in which this research was conducted, assumed power imbalances were believed to influence collaborative theory refinement. The introduction of theories or ideas was not often met with contradictions or substantial changes or elaborations from research participants. That is, the research participants’ answer was often the same as the researchers proposed idea, resulting in little theory refinement. Challenges in terms of constrained views and respondent bias were noted, highlighting that power imbalances may have influenced theory refinement.

### Challenge 1: recommendations

Addressing power imbalances within research, especially community-based research, involves many considerations throughout the research cycle.^35 36^ Within the interview phase specifically, the research team tried different techniques to mitigate effects arising from these imbalances. All interviews included a short discussion that did not focus on theory testing or refinement, conducted prior to theory testing components, but rather asked the participants about their experience with the programme or other related topics.^37^ During this, the research team asked more open-ended questions that were then used to help facilitate the more specific theory testing portion of the data collection. Direct questions related to the programme theories being explored were often posed before the more theory testing questions, as demonstrated by this excerpt from an interview guide from the second study (note, wording of questions and further insight into realist interviewing was informed by the ‘Realist Qualitative Interviewing’ lecture, facilitated by Dr Justin Jagosh within the Advanced Study in Realist Methodology course^38^):

Q: Do you think that the trainings have made you more confident in caring for a sick child?

Q: There is this idea that, the more confident you are your ability, the more likely you are to seek care from a health worker. What do you think about that?

Q: What is it about being confident that leads you to consult a health worker?

Notably, and as a consequence of the research context and translation (discussed below), the questions/theories put forth were presented as straightforward as possible. In some sites, visual diagrams depicting the theories were used but were met with mixed results. This was less successful when diagrams were not simplified enough. The challenges and the strategies we deployed to overcome these led to the development of key recommendations for managing power imbalances within realist evaluation interviews. Please see box 1 for a full list of recommendations.

Box 1 **Recommendations for reducing power imbalances**

- Follow traditional qualitative methodology interview techniques of building rapport, active listening, body language and so on.
- Use non-intimidating language and be mindful of how you introduce the conceptual structure of the interview.
- Incorporate an open interview phase before working towards theory testing/refinement with the realist interview.
- Consider using focus group discussions with groups that may be particularly influenced by power imbalances (if appropriate).
- Keep visuals or other tools (ie, examples, vignettes) simple and to the point.
- Be reflexive and document your interviews well, including if/how power may have influenced your interview. Ensure that you bring this into your analysis phase.

## Challenge 2: working through translators and across language

It is likely that conducting a realist evaluation in a cross-cultural context will require translation while interviewing some stakeholder groups. To date, very little methodological writing has explored realist data collection through translation. As RAMESES II Reporting Standards requires researchers to ‘describe the steps taken to enhance the trustworthiness of data collection and documentation’,^10^ this topic is timely. Conducting research outside of your language and/or culture without a properly managed translation process can result in missing important pieces of data or difficulty in discerning the CMOC elements. Appropriately managing the translation process requires both careful practical and ontological considerations. Ontologically, I considered translation in realist research, like the paradigm itself, to sit in the middle of constructionist (decoding meaning) and more positivist (direct translation). While it was important to have a record of the direct translation, these had to be reviewed with subjectivity in mind.

Managing translation was met with several challenges. As an ‘engaged realist researcher’ working to identify CMOCs, it was difficult to have the interviewer use the semistructured guide with little input from myself throughout the course of interview. After reflecting on some early transcripts, this process was amended. Moving forward, the interviewer would work through the guide, but then translate participant’s contributions to theory (whether they be contexts, mechanism, outcomes or CMOCs) when they arose so that together, we, as engaged realists, could further explore these important aspects with research participants. As such, the English parts of the recordings of each interview compared with the translated transcripts of that interview provided almost as much important explanatory information.

### Challenge 2: recommendations

Some of these challenges could be addressed through frequent communication and a constant feedback loop between researcher and translator/interviewer. In-depth methodological and tool training was required. Both translation and transcription needed to be followed by further unpacking meaning behind research participant’s responses and iteration became a necessity to ensure our understanding and interpretation of the data was accurate. Translators themselves constituted a separate research participant group; in-depth discussions were had on their understanding of theory refinement and CMOCs generated after interviews were conducted. Together, the teams reviewed the preliminary analysis and frequently discussed their programme theories. Being diligent on documenting reflexivity and decision-making processes also proved helpful, especially when reviewing transcripts at a later date.

It became clear that main considerations arising from translation within this work were less to do with eliminating bias (though not power, as discussed above) and keeping flow and more to do with ensuring that intended and unintended outcomes, and their generative causality, are captured and are contributing to theory refinement.^10^ Future realist work under similar conditions would benefit from a more intentional exploration of how best to conduct realist data collection through translation including consideration on how to incorporate translator’s understanding into the analysis of CMOCs and programme theories more robustly. Please see box 2 for a full list of recommendations.

Box 2 **Recommendations for translation**

- Plan for robust training on realist methods and realist interview techniques and allow for additional translation time.
- When translating from the local language to that preferred for analysis, be cautious of ‘direct’ translation alone. Theory refinement relies less on the exact wording, and more on the meaning and explanations. Make sure you can balance both of these.
- Consider translators as research subjects—after data collection is complete, interview them on their understanding of the intervention and theory refinement based on that interview.
- Team data analysis or at least feedback findings to translators or other research assistants.
- As with all realist evaluations, iteration becomes extremely important in when conducted through other language.
- Ensure diligent documentation of decision-making processes and reflexivity.

## Challenge 3: being ‘engaged’ with limited contextual familiarity

Realist evaluators are ‘engaged’ researchers—that is, we have an understanding of what is happening and why and use this to identify CMOCs and refine theories. It is advised, when possible, to have an understanding of the natural setting prior to interviews.^37^ It is, of course, impossible to know and understand any context in its entirety, but these challenges are exacerbated if the researcher is new to the research setting or a foreign researcher. This is often the case for global health research in LMICs and may be a particular challenge for realist evaluations. I believed, nevertheless, that during the studies presented here, I was a somewhat well-engaged realist researcher prior to commencement, having previously done work in Kabale (Uganda), having lived in Tanzania and having been seconded to the NGO in Kenya for 2 years. Yet, there were still many challenges in identifying context and mechanisms. Social context, including culture and tradition, power dynamics and relationships, require additional time and investment to begin to understand how they influence people’s reasoning and reactions when one is less familiar with such an environment. Without unpacking context and its influence on people in your setting, one can easily think that incomplete, or untrue, mechanisms are being triggered.

### Challenge 3: recommendations

Several authors have provided recommendations that can support increasing contextual understanding and/or relevance of a realist evaluation. Ebenso *et al*.’s recent work highlights how embedding a realist evaluation within existing Ministry of Health policies and practice facilitated collaboration between researchers and in-country policymakers and implementers.^24^ Involving such collaborators across all stages of the realist evaluation ensured study relevance and contextual significance,^24^ which could also contribute to the uptake of findings.^39^

The studies discussed herein took a number of steps to mitigate contextual misunderstanding at the onset. Following the suggestion by Adams and colleagues,^26^ the study designs included multidisciplinary partnerships to enable insights that go beyond technical recommendations. Clear communication channels were established to support the contribution by team members, and procedures were taken to ensure the engagement of programme decision-makers throughout the research process. Reflexivity was also built into the process which enabled methodological challenges to be identified and addressed throughout the study.^8 26^

While the above likely contributed to greater contextual significance, based on experiences in the first study, additional steps were taken in the second study, including a detailed gender analysis informed by the Research in Gender Ethics group RinGs.^40^ This analysis was used to support the theory gleaning phase, and together interviews for theory gleaning and the gender analysis worked to identify IPTs that are then tested through two subsequent rounds of data collection. Frequent referral back to the gender analysis during CMOC identification and theory refinement continues to highlight its importance. Using such a tool may also contribute to issues described in Challenge 1, as applying a feminist lens within research can be an important resource to understand and address power.^41^ The incorporation of one of the many tools used within research and development sectors to understand context (ie, contextual analysis, gender analysis, political analysis and so on) warrants further consideration and exploration within realist evaluation methodology.

Communication was also found to be an important tool in this regard. It is important once an initial understanding has been reached through reading and engaging with a particular context to discuss (both formally and informally) with others involved and not involved in the research process. Your ability to do this might be predicated on your relationships and length of time spent within the environment: do not underestimate the importance of both of these. Please see box 3 for a full list of recommendations.

Box 3 **Recommendations for contextual familiarity**

- Embed yourself in the research setting as much, and as long, as possible prior to and during study.
- Read outside of the specific discipline. While important for any realist research, might be especially important while working in an unfamiliar context.
- Have a diverse research support team who have insights into the context of study and ensure they are meaningfully incorporated across the realist evaluation cycle (including translators).
- Incorporate additional tools early on in theory elicitation, like gender analysis or contextual analysis.
- Read, watch, listen and then ask questions to friends and colleagues—it is amazing what you can learn with a little insight and some open dialogue.

## Challenge 4: programme theories—the detailed and the WEIRD

Many may fall victim to the desire to elicit IPTs with great detail and specificity; honestly, I did. However, when doing research in new or cross-cultural contexts, this could do your study a disservice. As per Challenge 3 above, if you are less familiar with the context of your research, IPTs (tentative theories proposed during early stages of the evaluation) may be misplaced. These early theories serve as the backbone for the rest of your study; poor IPTs will affect your subsequent activities which may mean missing out on some key elements and underlying theories. As summarised by Leeuw^42^ in relation to theories and interviewing, ‘to establish a dialectic atmosphere on the basis of empirically incorrect assumptions is inefficient and ineffective’.

This may be particularity important if formal theories are included, and specifically, WEIRD theories. WEIRD theories are those which have been developed through the study of ‘Western Educated Industrialized Rich Democratic’ individuals.^43^ Frequently belonging within the study of human behaviour and psychology, these more formal theories are often used as ‘universal’. Many question the ability of WEIRD theories to provide explanatory power to different contexts as they are developed through only studying ‘WEIRD’ people (whom are actually among the least representative populations).^44^ These broad claims on human psychology assume that there is little variation between individuals and populations; thus, a theory developed through the study of WEIRD people (often undergraduate psychology students) is applied to communities in rural Kenya without considerations for the people and context.

### Challenge 4: recommendations

Incorporating a theory gleaning phase into research design is recommended,^33^ and particularly encouraged when conducting work in a cross-cultural setting or less familiar context. After this, iteration should still be included which ideally results in having minimum of three rounds of primary data collection for gleaning, refining and testing,^33^ though such processes can occur within the same round of collection.^37^ If not including a theory gleaning phase, keep early theories to more broad concepts that provide space for meaningful refinement and bring WEIRD theories in during later rounds of refinement (if at all) or keep their use for examining your specific findings within a wider body of knowledge.

Within the first study, the socioecological model was used as an abstract conceptual framework to organise early candidate/initial theories, which proved very helpful. Using abstract conceptual frameworks has also been encouraged when evaluating ‘large complex and messy programmes’.^45 46^ This can allow for structure to be introduced and to draw important relationships between concepts, without requiring substantive theories early on. In evaluations with NGO partners, an intervention’s Theory of Change (if available) could provide a starting framework that is already designed around notions of how change will occur. The realist field could benefit from an exploration of efforts to use an intervention’s Theory of Change as a candidate or initial programme theory. Comparing, challenging and questioning WEIRD theories in relation to your findings can help to elevate our dependence on them within potentially inappropriate contexts, and these findings should be shared widely. Please see box 4 for a full list of recommendations.

Box 4 **Recommendations for theory**

- Include a theory gleaning phase, with minimum two subsequent rounds of iteration to refine theories.
- If no theory gleaning phase, keep early theories simple and allow for several iterative rounds of data collection.
- Avoid using WEIRD theories during early stages of your research.
- Bring WEIRD theories in to help explain your findings and situate them with wider the wider body of knowledge. If the findings allow, challenge or even refine WEIRD theories to suit your context.

## Conclusion

**Figure 2:**
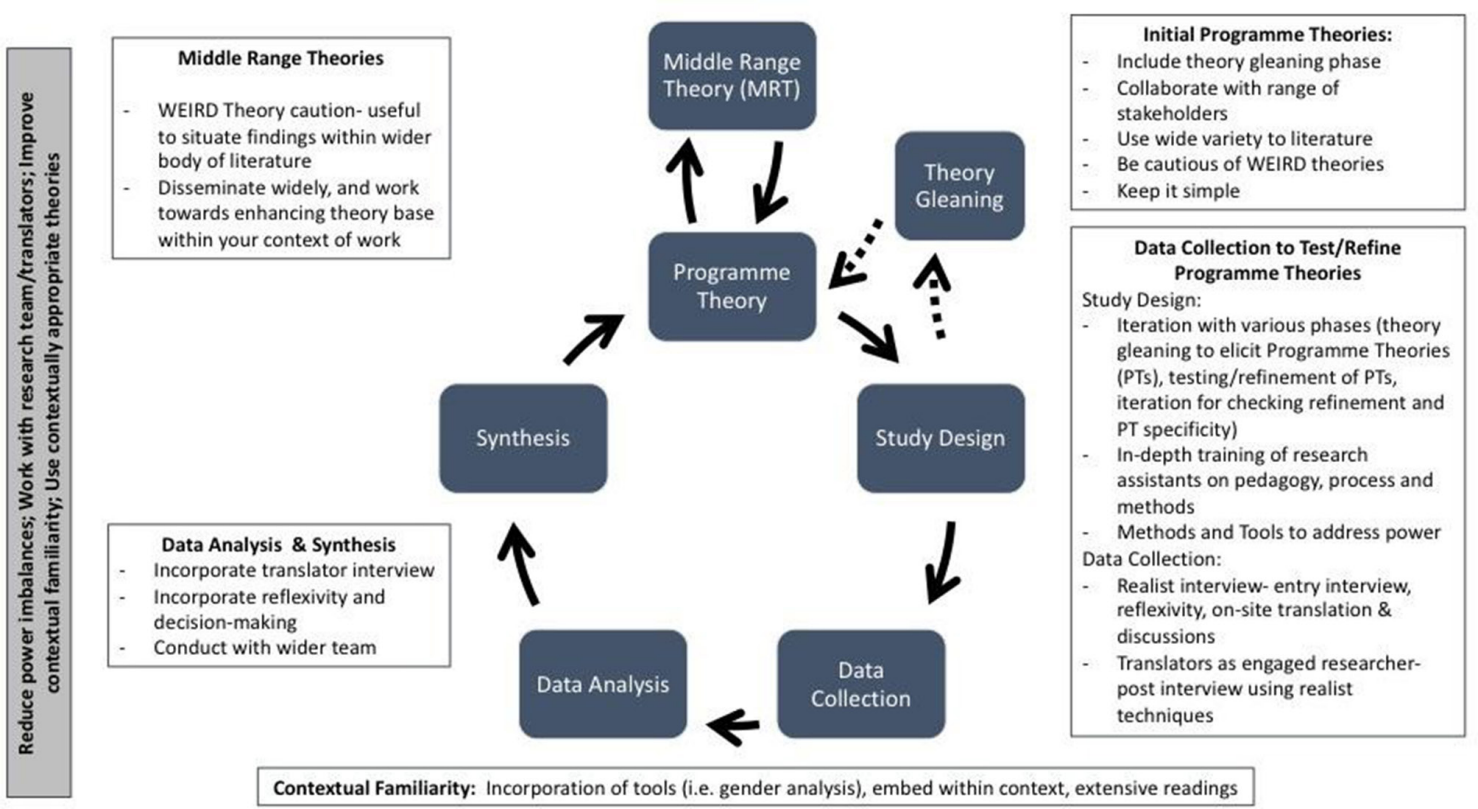
Realist evaluation cycle and considerations for cross context research. Realist cycle adapted from Mukumbang *et al* (2016).^48^

Realist evaluation methodology is being used within LMICs and cross-cultural contexts, yet little work explores this research process. Critical reflection of practices and knowledge generation within global health research is needed to enhance processes and work towards more relevant research that can have a positive impact on health.^32^ This paper presents some reflections and recommendations based on experience, but recognises the need for more detailed discussions on these topics, and for others to reflect on challenges and solutions they encountered while undertaking realist evaluations in general and within cross cultural contexts. Figure 2 summarises recommendations across the realist evaluation cycle noted within this paper.

Depending on the context and stakeholders, realist methods and tools might need careful consideration to address power imbalances. Exploring translators/translations within realist evaluations and how to conduct a realist interview to reduce power imbalances and promote theory refinement are especially encouraged. In less familiar contexts, considerations on how to ensure researchers can be properly engaged should be incorporated into the overall study design following examples set by Ebenso and colleagues^27^ and Adams and colleagues.^26^ This also aligns with continued calls for increased meaningful global health research collaboration.^39 47^

Particular attention should also be paid to early phases of the realist evaluation, leaving space for either theory gleaning or keeping theories simple. WEIRD theories should be used with caution, and researchers should disseminate their middle range theories widely so that there are more contextually specific theories others can draw from, to work to break the WEIRD conundrum. For realist evaluations within NGO interventions, exploring synergies between the intervention’s Theory of Change and realist candidate programme theories is recommended. Capacity strengthening for realist evaluation within such contexts is encouraged. Given that many realist evaluations in LMICs are being done for NGO programming to improve design and implementation, how best to ensure findings contain well refined theories that also have concrete and practical recommendations for action warrants further consideration.

These discussions need to continue, given the increasing interest from both academics and practitioners to use realist evaluation methodology within global health research and evaluations. Ensuring rigorous research that is responsive to the conditions in which it is being conducted will improve methodological rigour and make for more useful, reflective and accurate programme findings and recommendations.
